# Praziquantel treatment coverage among school age children against Schistosomiasis and associated factors in Ethiopia: a cross-sectional survey, 2019

**DOI:** 10.1186/s12879-020-05519-0

**Published:** 2020-11-23

**Authors:** Yilma Chisha, Zerihun Zerdo, Mekuria Asnakew, Chuchu Churko, Manaye Yihune, Abinet Teshome, Nebiyu Nigussu, Fikire Seife, Birhanu Getachew, Markos Sileshi

**Affiliations:** 1 College of Medicine and Health Science, School of public health, Arba Minch University, Arba Minch, Ethiopia; 2 College of Medicine and Health Science, collaborative research and training center for Neglected tropical diseases, Arba Minch University, Arba Minch, Ethiopia; 3 School of public health, Arba Minch University, College of Medicine and Health Science, Arba Minch, Ethiopia; 4 Department of Bio-medical science, Arba Minch University, College of Medicine and Health Science, Arba Minch, Ethiopia; 5 Federal Ministry of Health (FDRE, MOH), Addis Ababa, Ethiopia; 6 Federal Ministry of Health, Ethiopian public health institute (EPHI), Addis Ababa, Ethiopia

**Keywords:** Ethiopia, Mass drug administration, PZQ, Preventive chemotherapy, SCH, SAC, Coverage validation survey

## Abstract

**Background:**

World Health Organization estimated that 779 million people are at risk of getting schistosomiasis (SCH) and 240 million people were infected worldwide. SCH due to *Schistosoma mansoni* (*S. mansoni)* is a wide public health problem in Ethiopia. The aim of the survey was to quantify national and district disaggregated treatment coverage status for SCH and compare validated coverage with the one reported.

**Methods:**

Community based cross-sectional survey was conducted in April 2019 among households with school age children (SAC) 5–14 years in seven purposively selected districts of the country. Segments to be surveyed were randomly selected and households to be interviewed from each segment were determined using systematic sampling technique. A total of 3378 households visited and 5679 SAC (5–14 years) were interviewed.

**Results:**

Overall reported treatment coverage of Praziquantel (PZQ) against SCH was 4286 (75.5%). Males were 27% more likely to swallow the drug (AOR = 1.27; 95% CI: 1.09, 1.47) than females. SAC with age 10–14 years were 45% more likely to swallow the drug compared with their counter parts (5–9 years), (AOR =1.45; 95% CI: 1.25, 1.69). There is statistically significant association between PZQ swallowing status with school enrollment. (AOR = 20.90, 95% CI: 17.41, 25.08). Swallowing status of PZQ against SCH significantly higher for SAC treated in districts applied integrated treatment approach (87.5%) compared with SAC treated in vertical treatment approach (72.5%); *P*-value < 0.001. SACs were asked for reasons for not taking the drug and the main reported reason for not swallowing PZQ in the present study was none attending of the school.

**Conclusions:**

Over all treatment coverage of PZQ against SCH in the present study was 75.5%. Although it is in accordance with WHO recommendation for Ethiopia, national programmatic improvements are necessary to achieve higher coverage in the future. To increase treatment coverage for PZQ against SCH in Ethiopia, school based training should target all schools. Moreover, mobilization, sensitization and implementation of the community wide treatment need to be improved.

**Supplementary Information:**

The online version contains supplementary material available at 10.1186/s12879-020-05519-0.

## Background

Schistosomiasis (SCH) is a chronic water- related parasitic disease caused by blood flukes of the *genus schistsoma*. It is the most important helminthes infection in tropical developing countries in terms of its public health and socioeconomic importance [1]; Ethiopia is not exceptional. People become infected when coming in contact with water containing *schistosom*e infected snails [2] WHO report estimated, 779 million people are at risk of getting SCH infection worldwide, 240 million infected cases and more than 200,000 deaths occurring each year [3]. About 224 million of infected people live in Sub-Saharan Africa (SSA) [4].

SCH is the second most important parasitic infection: killing an estimated 280,000 people per year in African region [5] and in many parts of Ethiopia, SCH due to *Schistosoma mansoni (S.mansoni) and schistosoma haematobium (S. haematobium)* is a wide public health problem and usually occurs in agricultural communities living along small streams, irrigation schemes and lakes in altitudes below 2000 m above sea level [6, 7].

National Master Plan for neglected tropical diseases (NTDs) reported that 37.3 million people are living in SCH prone areas, comprising 3.4 million pre-school age children (PSAC), 12.3 million school age children (SAC), and 21.6 million adults [8]. The finding of systematic review, and meta analysis on SCH showed that the national pooled prevalence of *S. mansoni* infection among Ethiopian population was 18.7% (95% CI: 14.7–23.5). Other study in Northwest Ethiopia revealed that the prevalence of *S. mansonia infection* among PSAC was 11.2% [9].

Studies conducted in Amhara region, Ethiopia, revealed that the prevalence of *S. mansoni* infection among SAC was 89.9% in Sanja general elementary school [10], 82.8% in Sanja and Ewukat Amba primary school [11], 56.6% in Tach Armachio district [12], 37% in Zarima town of Gonder [13], 20.6% in Gorgora town [14], 14.3% in Ethiopian Orthodox church students around Lake Tana [15] and 2.8% in Bahir Dar Shimbit elementary school [16].

Different factors can be associated with *schistosome* infection; a study finding from south west Ethiopia revealed that the intensity of *S. mansoni* infection was significantly higher in males than females. Infection intensity of *S. mansoni* was also significantly higher among children attending school. However, intensity of *S. mansoni* infection was not significantly different on age categories [17].

The prevalence of schistosoma infection was increased with increasing age (χ2 = 61.8, *P* < 0.00). Reported history of lake visits (AOR = 2.31, 95% CI: 1.06–5.01, *P* < 0.03) and the proximity to the lake shore (< 500 m) (AOR = 2.09, 95% CI: 1.05–4.14, P < 0.03) were significantly associated with *S. mansoni* infection [18].

“Many studies in Ethiopia [10–16] revealed for high prevalence and intensity of *s.mansoni* infection among SAC. They also revealed human contaminative activities such as open field defecation and exposure activities such as washing, swimming and bathing in contaminated water bodies favors transmission of SCH. SAC are easily infected with *schistosoma* due to their frequent contact to the contaminated water bodies. SCH is devastating NTDs especially for resource limited settings with poor sewage disposal and in adequate supply of clean water. Therefore, to reduce the burden of SCH, WHO designed strategy for PZQ mass drug administration (MDA) and set goals to control and eliminate SCH by 2020 and 2025 respectively. In order to help the achievement of WHO goals, Ethiopia has started MDA of PZQ preventive chemotherapy (PPC) for all enrolled and non enrolled SAC in November 2015 in collaboration with Imperial College of London.

Therefore, the purpose of conducting the present coverage validation survey was to quantify national and district disaggregated treatment coverage and to compare validated coverage during the survey with the one reported by independent monitors (IM) during the actual drug distribution. It also aimed to identify possible challenges which might hinder effective implementation of MDA program to control and eliminate the infection of SCH in Ethiopia.

## Methods

### Study design, period and setting characteristics

Community based cross-sectional survey was conducted in April 2019 among households with SAC (5–14 years) in seven purposively selected districts of the country. Ethiopia is administratively classified into nine regions and two city administrations. According to the 2018 demographic profile, there are 105, 350,020 populations [19]; of which 51% is females, 47.3% were within the age range of 0–14 years. The total area of the country was 1104, 300 km^2^ [20] and more than 80% of the population were live in rural areas [19]. Looking to the weather condition of the country, Ethiopia has highland, midlan and lowland type of weather conditions.

The health policy of Ethiopia was preventive with a three tier health care delivery system (Primary health care, zonal hospital and referral or teaching hospital structure). The commonest public health problems to the populations of Ethiopia are communicable disease like malaria, tuberculosis, HIV/ AIDS, helmintheasis, chronic non-communicable disease. *S.mansoni* is among the commonest parasitic infections in Ethiopia.

**Fig. 1:**
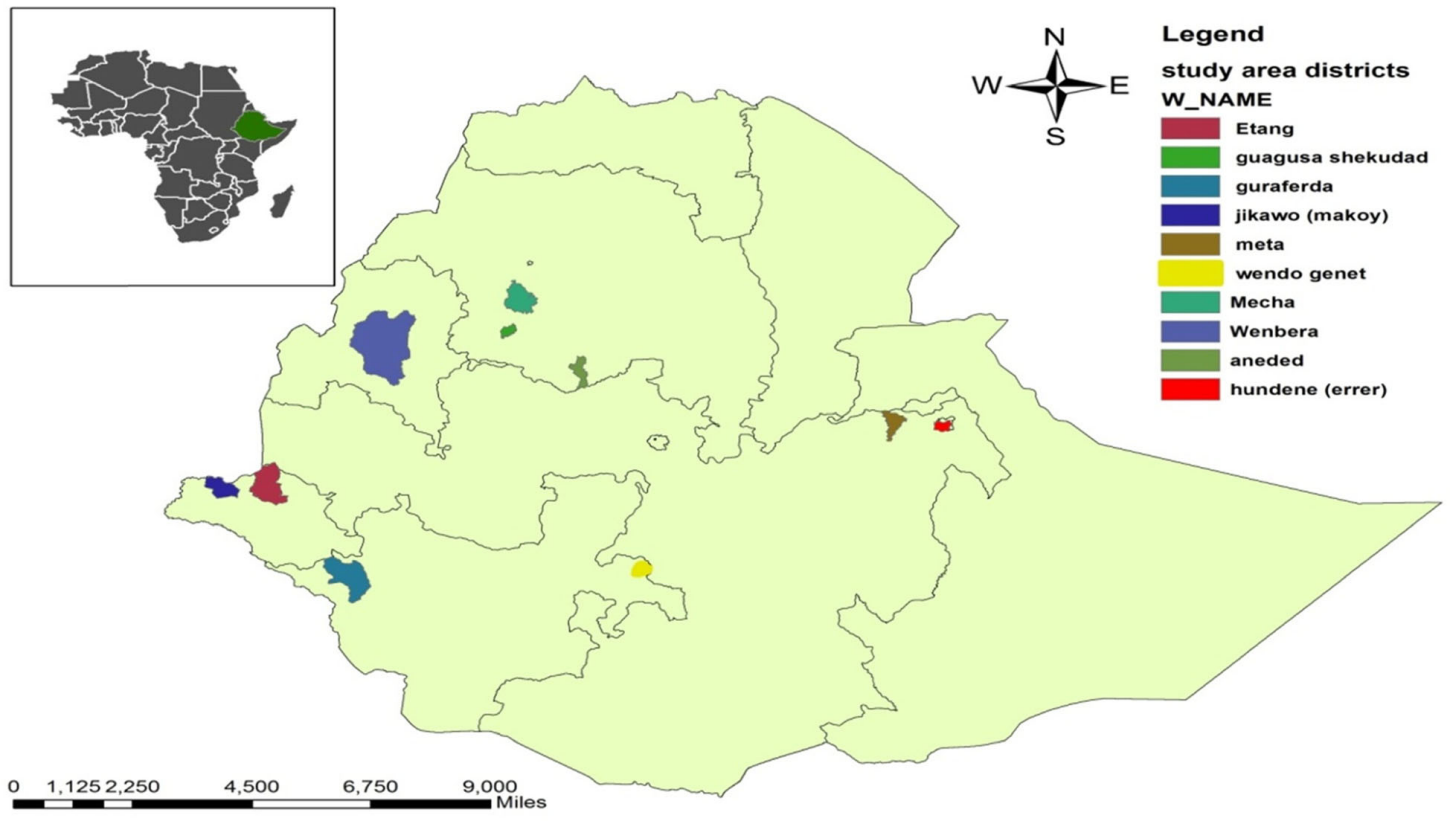
Map of study districts in which coverage validation survey was conducted, Ethiopia, 2019, (*Note*: it is an orginal scale map, prepared by the research team using GPS data).

This study was conducted in seven purposively selected districts of the country. Districts were selected based on their case report of SCH. Lists of regions with their respective districts: Errer district from Harar region, Ittang special from Gambella region, Mecha from Amhara region, Meta from Oromia which is the largest region with the highest number of population in the country, Gura ferda and Wondogenet from Southern Nations, Nationalities and People (SNNP) region and Wombera from the Benshangul Gumuz region. In each of the districts, the total population is more than 100,000 (Fig. 1).

### Survey procedure, sample size determination, analysis and presentation of findings

One month prior to the MDA campaign, woreda health officers in each district were trained on how to cascade the deworming process and to train health care workers and teachers. Two weeks before administering PZQ to the SAC, two teachers from each school and one health professional from respective kebele (smaller administrative units of a district) was trained for 3 days by woreda health officers. Lists for enrolled SAC to be treated were taken from school register, while non-enrolled SAC were treated by doing community mobilization to come to the nearest treatment center. The number of PZQ tablets to be administered was determined using WHO dose pole. The MDA campaigns have been conducted 1 month prior to the present coverage validation survey.

The drugs were distributed by health care professionals and community health workers. The SAC (both enrolled and non-enrolled) were swallowed the medication in front of the drug distributor after informing all the necessary drug use indications. One month prior to the current coverage validation survey, all the required information’s was observed, interviewed and recorded by independent monitors (IM). Therefore, the present coverage validation survey was done to validate the number of SAC who were treated in the last MDA campaign 1 month preceding this survey which was reported by IM. To perform this coverage validation survey, we used the sentinel site list from Federal Ministry of Health (FMOH), then seven districts purposively selected from six regions of the country: five districts undertake vertical treatment approach while two were undertaken an integrated.

The number of segments to be surveyed in each district was identified by coverage survey builder. Randomly selected segments should contain at least 16 households and the final participant households to be interviewed were selected by systematic random sampling technique after getting selection interval by dividing 50 to 16; which is K = 3. Using the above sampling procedure, from seven districts a total of 3378 households were visited and 5679 SAC were interviewed to validate treatment coverage of PZQ against SCH in Ethiopia.

Data was collected using mobile phone data collection application of SurveyCTO. At the end of data collection, it was further transferred to STATA statistical analysis soft ware StataCorp version 14 for cleaning and analysis. Descriptive statistics was/were done and presented by table, graphs and text narration. Cross tabulation and binary logistic regression analysis was also done. Bivariate and multivariable binary logistic regression analysis was done to select potential candidate variables and to estimate the independent effect of predictors on swallowing status of PZQ and to control potential confounders’ respectivelly. Variables which satisfied the *p*-value criteria of <=0.25 in bivariate logistic regression analysis were taken as candidate variables for multivariable logistic regression analysis (Table 5). Model was built using step wise backward elimination model building procedure and the effect of using model with reduced or many variables were compared by log likely hood ratio test. The instability of regression coefficient (Multicollinearty) was checked using Variance inflation factor (VIF) and the cutoff point was mean VIF greater than 10 to have significant collinearty.

**Fig. 2:**
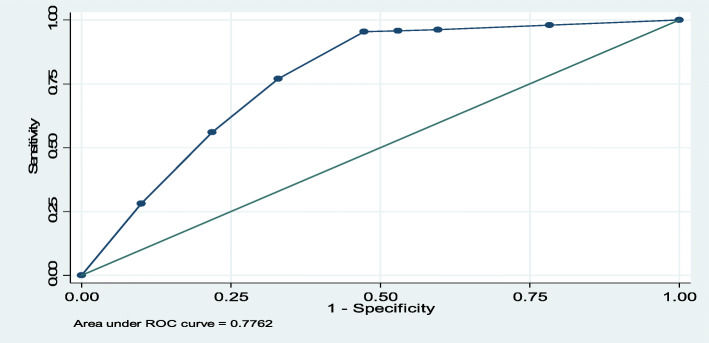
ROC curve showing prediction performance of variables in the final fitted model on swallowing or acceptance of PZQ among SAC, sampled districts, Ethiopia, 2019

The classifying ability or prediction performance of variables in the final fitted model was checked using Receiver Observed Characteristics (ROC) curve and 77.62% of PZQ swallowing was determined by SAC age, gender of SAC and school enrollment status of SAC (Fig. 2). The association between dependant and independent variables was measured by AOR and statistical significance was assured by *P*-value < 5% (< 0.05). Ethical approval to conduct the current survey was obtained from FMOH, Arba Minch University, and verbal consent was obtained from the guardian of the child.

## Results

### Socio-demographic characteristics of study participants

Among 5979 SAC (5–14 years) eligible for interview in seven districts, 5679 SAC were interviewed, yielding a response rate of 95%. The mean age of SAC was 9.6 years with standard deviation of ±2.8. Almost males and females were equally involved in the survey. Concerning to the school attendance, 4750 (83.6%) children attended school; of which 4732 (99.6%) children attended at primary school. Majority, 4612 (97.1%) children attended school which was owned by public (Table 1).

**Table 1:** Socio-demographic characteristics of SAC in study districts, Ethiopia, 2019

S. No	Variables	Categories	Frequency	Percent (%)
1	Age (Years)	5-9	2765	48.7
		10-14	2914	51.3
2	Gender	Female	2853	50.2
		Male	2826	49.8
3	School Attendance	No	929	16.4
		Yes	4750	83.6
4	Educational level	Primary	4732	99.6
		Secondary	18	0.4
5	School type	Public	4612	97.1
		Private	126	2.6
		Religious	12	0.3

### PZQ treatment coverage against SCH among SAC in study districts, Ethiopia

Out of 5679 interviewed SAC in districts undertake vertical and integrated mass drug treatment approach, the overall treatment coverage of PZQ against SCH was 75.5%.

**Fig. 3:**
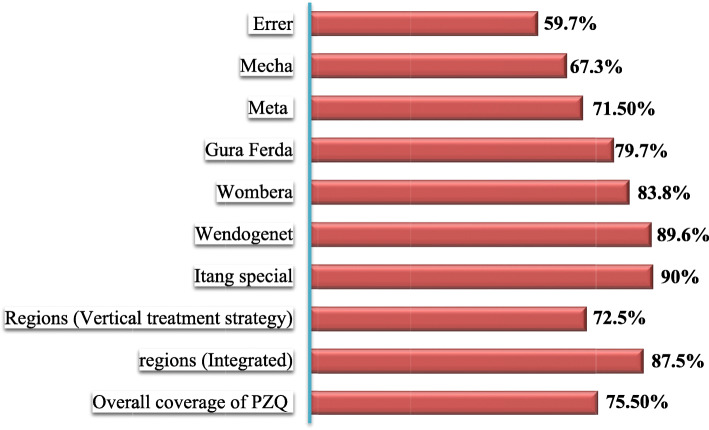
District disaggregated treatment coverage of PZQ among SAC, in the sampled districts of Ethiopia, 2019.

From 4571 interviewed SAC in districts undertake vertical treatment approach, 3316 (72.5%) of SAC reported that they were treated with PZQ; and among 1108 SAC interviewed in districts undertake integrated treatment approach, 970 (87.5%) were treated with PZQ against SCH. District disaggregated treatment coverage of PZQ showed that students from Itang special in Gambella region reported the highest treatment coverage (90%) and SAC from Errer district in Hareri region had the lowest (59.7%) treatment coverage (Fig. 3).

### Treatment coverage of PZQ disaggregated by gender, age, school attendance and heard MDA in the sampled districts, Ethiopia, 2019

Gender disaggregated treatment coverage of PZQ showed that males were significantly more likely to swallow the drug (77.6%) than females (73.3%) (X^2^ = 14.77, *p*-value< 0.001); treatment coverage of SCH was significantly different between age groups of SAC. SAC with 10–14 years swallowed the drug better (83.7%) than 5–9 years (66.8%) (X^2^ = 222.66, *p*-value <=0.001). Statistically significant difference was observed in swallowing status of PZQ among SAC who attended school and heard about MDA when compared with their counter parts (Table 2).

**Table 2:** Treatment coverage of PZQ among SAC disaggregated by gender and other predictors in the sampled districts of Ethiopia, 2019

Variable	Categories	Swallowed PZQ						Chi-square (*p-*value)
		Yes		No		Unknown		
		No	%	No	%	No	%	
Gender	Female	2092	73.3	679	23.8	82	2.9	14.77 (0.001)
	Male	2194	77.6	556	19.7	76	2.7	
Age (Years)	5-9	1848	66.8	799	28.9	118	4.3	222.66 (0.001)
	10-14	2438	83.7	436	15	40	1.4	
School attendance	Yes	4091	86.1	616	13	43	0.9	1833.40 (0.001)
	No	195	21	619	66.6	115	12.4	
Heard about MDA	Yes	413	91.3	371	8.4	9	0.2	2696.39 (0.001)
	No	271	21.1	864	67.3	149	11.6	

### SCH treatment coverage disaggregated by school attendance in districts undertook vertical treatment approach

The school attendance disaggregated analysis in districts conducted vertical treatment approach showed that PZQ treatment coverage among SAC was significantly higher for SAC who attended school (84.1%) compared to their counter parts (14.4%) (X^2^ = 1595.32, *p-*value< 0.001) (Table 3).

**Table 3:** Treatment coverage of PZQ disaggregated by school attendance and other predictors for districts applied vertical treatment approach, Ethiopia, 2019

Variable	Categories	Swallowed PZQ						Chi-square (*p-*value)
		Yes		No		Unknown		
		No	%	No	%	No	%	
Gender	Female	1624	70.2	614	26.6	75	3.2	12.97 (0.002)
	Male	1692	75.0	500	22.1	66	2.9	
Age (Years)	5-9	1405	63.4	709	32	103	4.6	186.19 (0.001)
	10-14	1911	81.2	405	17.2	38	1.6	
School attendance	Yes	3207	84.1	566	14.9	39	1.0	1595.32(0.001)
	No	109	14.4	548	72.2	102	13.4	

### Treatment coverage of PZQ among districts involved vertical and integrated treatment approach

Swallowing status of PZQ against SCH significantly higher among SAC in districts conducted integrated treatment approach compared with vertical (Table 4).

**Table 4:** Treatment coverage of PZQ disaggregated by treatment approach, Ethiopia, 2019

Treatment approach	Swallowed PZQ			X^2^	*P-*value
	Yes (%)	No (%)	Unknown (%)		
Vertical	3316(72.5)	1114(24.4)	141(3.1)	108.5	<0.001
Integrated	970(87.6)	121(10.9%)	17(1.5%)		

### Reported reasons for not swallowing PZQ among SAC in study districts, Ethiopia

**Fig. 4:**
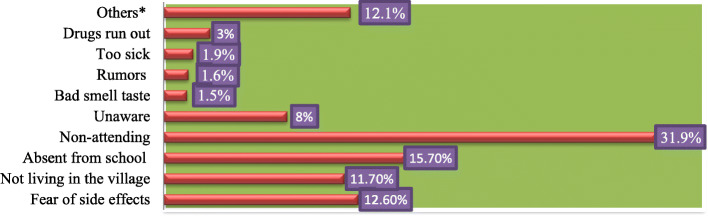
Professed reasons for not swallowing PZQ among SAC in study districts of Ethiopia, 2019. **Key**: Others*: not definite reasons stated, too far, no MDA, not eaten food, not invited

The main reported reason for not swallowing PZQ against SCH among SAC was not attending school (*n* = 394, 31.9%) (Fig. 4).

### PZQ distribution sites reported by interviewee

**Fig. 5:**
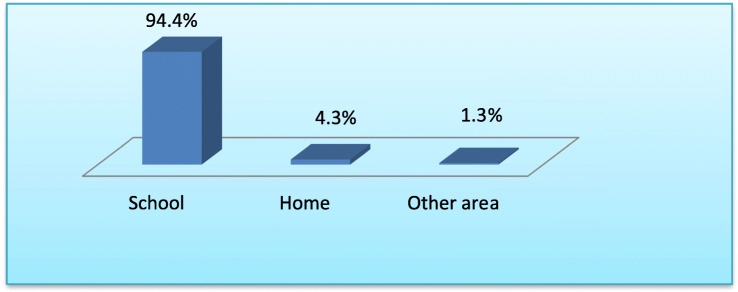
Distribution sites of PZQ against SCH in selected districts of Ethiopia, 2019. **Key**: Others*: Village head house, Central point, Local health center

Most of interviewed SACs in study districts reported that they had received PZQ treatment against SCH from school, 4044 (94.4%) **(**Fig. 5).

### Factors statistically and significantly associated in multivariable logistic regression analysis

Multivariable logistic regression analysis showed that age of SAC, gender and school enrollment status of SAC were statistically and significantly associated with PZQ acceptance and swallowing. Students with age 10–14 years were 45% more likely to accept and swallow PZQ mass treatment compared with their counter parts (5–9 years); (AOR = 1.45, 95% CI: 1.25, 1.69).

After stabilizing age and school attendance in the model constant and compare males with females towards PZQ swallowing status, males were 27% more likely to accept and swallow PZQ compared with their counter parts. (AOR = 1.27, 95% CI: 1.09, 1.47); Attendance to the school was found to be statistically and significantly associated with PZQ swallowing against SCH. SACs enrolled to private or public school were almost 21 times more likely to swallow PZQ compared with SACs who didn’t; (AOR = 20.90, 95% CI: 17.41, 25.08) (Table 5).

**Table 5:** Factors statistically and significantly associated with PZQ swallowing in multivariable logistic regression analysis among SAC in the sampled districts of Ethiopia, 2019 (*n*=5679)

Variable	Category	Swallowed PZQ		COR	***P-***value	AOR	95% CI
		Yes N (%)	No N (%)				
**Age**	5-9 year	1,848 (43.1)	917 (65.83)	1	0.000	1	**-**
	10-14 year	2,438 (56.9)	476 (34.17)	2.54		1.45	**(1.25, 1.69)
**Gender**	Female	2,092 (48.81)	761(54.63)	1	0.000	1	**-**
	Male	2,194 (51.19)	632 (45.37)	1.26		1.27	*(1.09, 1.47)
**School attendance**	No	195 (4.55)	734 (52.69)	1	0.000	1	-
	Yes	4,091(95.45)	659 (47.31)	23.37		20.90	**(17.41, 25.08)

*COR* Crude Odds ratio, *AOR* Adjusted Odds Ratio, *CI* Confidence interval, *= *P* value < 0.05, ** < 0.01

To check the classifying or prediction performance of variables in the final fitted model, ROC curve was done. Based on the Hosmer and Lemeshow criteria, variables in the fitted model were predicting the acceptance or swallowing of PZQ by 77.62%.

This means 77.62% of PZQ swallowing among SAC was determined by age of SAC, gender of SAC and school attendance or enrollment status of SAC (Fig. 5).

### Model equation for the final fitted multivariable logistic regression analysis

$$ \ln\;\left[\frac{\mathrm{p}}{1-\mathrm{p}}\right]=\upalpha +{\upbeta}_1{\mathrm{x}}_1+{\upbeta}_2{\mathrm{x}}_2 $$

0.22+ 0.375* age of SAC + 0.237*gender of SAC+ 3.04*school attendance (Table 5)

## Discussion

There has been considerable progress towards WHO Roadmap goals for SCH and regional targets, as the control of SCH has become a priority on the agenda of many governments. This drive has encouraged many countries to establish programmes and develop national action plans to control NTDs. By 2016, 36 African countries had developed and launched their national NTDs master plans. Ethiopia is among the countries struggling to control and eliminate the infection of SCH in Africa.

The current survey validation revealed that over all coverage of PZQ acceptance and swallowing rate among SAC against SCH was 75.5%. This finding is almost consistent with the study finding in Mali, coverage rate of 76.7% [21]; but it is low compared with the pilot treatment given by WHO (2008–2009) one million school children were treated in the selected schools with 86% PZQ coverage [22] and 86.9% PZQ treatment coverage in Zanzibar [23]. Possible reasons for this inconsistence might be variation on sample size and target population. In our study only SAC participated whereas in WHO and Zanzibar study both SAC and adults were almost equally involved. It is obvious that adults can accept and swallow the drugs more effectively than children’s as the drugs were large in size and not convenient for children to swallow.

For Ethiopia, the target coverage level for SCH that was recommended by WHO was 75%; hence the current finding was in line with WHO recommendation of 2011. Achieving target MDA coverage level for SCH is essential to eliminate and control SCH. To ensure program goals are met, coverage level reported by drug distributors may be validated through household coverage surveys that rely on respondent recall.

According to the study finding in Ivory Coast, overall PZQ treatment coverage rate was 47.6% (2730/5733). It also revealed that treatment compliance (acceptance and swallowing) status of PZQ was significantly higher in males than females (X^2^ = 19.96, *p* < 0.001) [24]. This finding is consistent with the present study finding, the odds of acceptance and swallowing of PZQ is 27% more likely for males compared with their counter parts.. A study finding in Uganda revealed that over all PZQ treatment coverage was 48.8%. Factors that improved individuals’ odds of taking PZQ were school enrolment and age of children [25].

This finding is consistent with the finding of the present study; the odds of swallowing PZQ for school enrolled SAC almost 21 times more likely compared with SACs not enrolled to school. Similarly, SACs with in age category of 9–14 years were 45% more likely to accept and swallow the PZQ than SACs within age category of 5–9 years (Table 5). The most frequent reasons for not taking the 2016 PZQ distribution in Uganda were not being drugs offered and being away during MDA [25]. Other study by Knopp and colleagues in Zanzibar revealed that, the main reported reasons for not receiving or taking PZQ were: absence during drug distribution, not drug distributor reached the household, fear of adverse events, being pregnant, and breastfeeding or feeling unhealthy [23]. These findings were almost similar with the reasons reported for not to swallow PZQ in the present study (Fig. 3).

### Policy implications

SCH is related with low socio-economic condition, poor hyigine and sanitation, lack of access to healthcare facilities and recurrent contact with contaminated water sources [25, 26]. Offering of MDA two times per year has been successful at dropping down the magnitude and intensity of infections. Even though Preventive Chemotherapy (PC) is a significant component of SCH control, other helpful strategies will also be considerd for the control and elimination of the diseases.

### Strengthen and limitations of the study

This study has important strengths and limitations. As strength: it directly measured the accuracy of respondent recall in the setting of MDA. It provided an opportunity to investigate respondent recall not only for overall MDA participation, but also for recall of specific medications swallowed by showing the actual drugs. This survey tried to gather data by repeat visit in case the household and SAC was not present at home. Perceived limitations of this study will be: interviewing the SAC for the MDA campaign conducted before a month might have possibility of recall bias. Due to cross-sectional study design, can’t measure casual inference and temporality of sequence.

## Conclusion

The present study showed that overall treatment coverage rate of PZQ against SCH among SAC in Ethiopia is 75.5%; it is in line with WHO recommendation (75%). The present study also revealed that female SACs, non enrolled SAC, SACs who were not heard for the undertaking of MDA campaign and SACs within age category of 5–9 years were less likely to swallow PZQ against SCH compared with their counter parts. Although self-reported MDA coverage was in accordance with WHO recommendation, national programmatic improvements are necessary to achieve higher coverage. Adequate community mobilization and improved training for MDA distributors, monitors and supervisors should be given to improve PZQ treatment coverage in the future MDAs.

### Recommendations

Community mobilization approaches need to be strengthened in order to reach non-attending or non-enrolled school-aged children. To increase coverage and compliance of PZQ in Ethiopia, school based training (SBT) should target all schools and mobilization, sensitization and implementation of the Community Wide Treatment (CWT) need to be improved. To attain elimination strategy of SCH, a very high treatment coverage and compliance of PZQ at national, regional and local level is a key and additional control measures such as biological snail control and behavioral change interventions will need to be considered. The benefit of MDA and drug side effects should also be clearly communicated toSAC and the drugs should be broken in to two or more parts to easily facilitate swallowing for the SAC especially for those bellow 10 years.

## Supplementary Information

**Additional file 1.**

**Additional file 2.**

## Data Availability

The dataset analyzed for this study is available from the corresponding author (Yilma Chisha Dea) on reasonable request. You can access the data set used for analysis which is attached as annex document in the manuscript tracking system.

## Abbreviations

CWT: Community Wide Treatment
FMOH: Federal Ministry of Health
MDA: Mass Drug Administration
NTDs: Neglected Tropical Diseases
PC: Preventive Chemotherapy
PSAC: Pre-school age children
PZQ: Praziquantel
SAC: School Age Children
SBT: School Based Training
SCH: Schistosomiasis
SSA: Sub-Saharan Africa
WHO: World Health Organization
